# Prescription Department

**Published:** 1893-12

**Authors:** 


EXPECTORANT FOR ACUTE BRONCHITIS.

℞. Ol. terebi th. ʒij-iij.
Muc. accaciæ, q. s.
Aq. cinnamon, ℥j.
Aquæ, q. s. ad, ℥vj.

M. Sig.—Tablespoonful in water every four hours.

GONORRHŒA.

Gargiano recommends the following formulæ:

℞. Acid. carbol. pur., gr. viii-xvj.

Glycerin., fʒi½–iiss.

Aq. dest., f℥ix.

M. Sig.—Inject three or four times a day.

℞. Acid. carbol pur., gr. iv–ʒss.

Zinci sulphat., gr. viii–xvj.

Glycerin., fʒiiss–f℥ss..

Aq. dest., f℥ix.

M. Sig.—For injection.

In chronic cases:—

℞. Acid. carbol., pur., gr. viii–xvj.

Zinci sulph., gr. xxiv–ʒss.

Glycerin., fʒiiss–℥j.

Aq. dest., ℥xviij.—M

—*El Siglo Medico.*

CARDIAC TONIC.

Dr. Edward C. Mann, of Brooklyn, N. Y. in the *Physician and Surgeon* gives the following formula:—

℞. Caffein, hydrochlor., ʒiss.
Strychniæ sulph., gr. j.
Extract. belladonnæ, gr. iss.
Fel bovis insp., ʒj.
Ext. colocynth. comp., gr. xxx.
Extract. aloes, gr. xxx.
Extract. taraxaci, gr. xlv.

Misce et fiant pil. no. xxx.

Sig.—One three times a day.

NASAL CATARRH.

Professor Chas. A. Wilson, of St. Louis, frequently uses the following, which can be varied to suit the case:—

℞. Liq. alboline, ℥ij.
Oil eucalyptus, gtt. x.
Terebene, ʒss.
Menthol, gr. v.
Oil gaultheria, q. s. to scent.

Use in compressed-air spray.—*St: Louis Clinique*

FOR COUGH.

℞. Antikamnia ʒj.

Salol.,

Quin. sulph., aa gr. xx.

Spts. frumenti, ℥iij.

Syr. Tolutan., ℥j.

Syr. Simplex, q. s, ℥vj.

M. Sig.—One teaspoonful every hour until the cough is relieved.—*Times and Register.*

CHRONIC RHEUMATISM.

The following appears in the *Med. Bulletin:*

℞. Tinct. Guaiaci eth., ℥j.

Tinct. Cannabis Indicæ eth., ʒvj.

Tinct. Colchici eth., ʒij.

M. Sig.— Twenty-five to thirty drops on sugar every four hours.—*Hospital Gazette.*

ANAL FISSURE.

Allingham strongly advocates the local use of the following ointment:—

℞. Hydrarg. subchlor., gr. iv.
Pulv. opii,
Ext. belladonnæ, aa gr. ij.
Ung. sambuc., ʒj.

M. Sig.—To be applied frequently.

He states that he has had many cures with this ointment alone. Another excellent ointment, recommended by the same authority, is:—

℞. Plumb. acetatis,
Zinci oxidi aa gr. x.
Pulv. calaminæ, gr. xx.
Adipis benzoinat., ℥ss.—M.

An ointment of the oxide of mercury, 30 grains to the ounce, has cured many cases.—*Doctors' Weekly.*

CHRONIC DIARRHŒA.

Dr. W. C. Buckley, of Philadelphia, has used with success the following:—

℞. Pulv. ipecacuanhæ, gr. x.

Pulv. populvs trem., ʒj.

Pulv. capsici, ʒiss.

Pulv. xanthoxylum, ʒj.

Pulv. myrica cerif., ʒj.

Mix and make into four grain pills.

